# Co-occurrence of anti-phospholipid syndrome and posterior reversible encephalopathy syndrome in a patient with Autoimmune hepatitis: A case report

**DOI:** 10.1016/j.radcr.2024.06.078

**Published:** 2024-07-27

**Authors:** Ghazaleh J. Soufi, Seyed H. Tooyserkani, Ali Hekmatnia, Ali Norouzi, Amirhossein Sadeghian, Farshad Riahi

**Affiliations:** a Department of Radiology, Isfahan University of Medical Sciences, Isfahan, Iran; b School of Medicine, Isfahan University of Medical Sciences, Isfahan, Iran; c Professor of Radiology, Isfahan University of Medical Sciences, Isfahan, Iran; d Department of Radiology, Alzahra Hospital, Isfahan University of Medical Sciences, Isfahan, Iran; e Department of Radiology, Zabol University of Medical Sciences, Sistan and Baluchestan Province, Iran

**Keywords:** Posterior reversible encephalopathy syndrome, Autoimmune hepatitis, Antiphospholipid syndrome

## Abstract

Posterior reversible encephalopathy syndrome (PRES) is a neurological disease characterized by a variety of neurological findings, in accordance with radiological characteristics. PRES is commonly secondary to elevated BP and/or conditions such as autoimmune patients receiving immunosuppressive drugs. Our case involves a 36-year-old female with a history of autoimmune hepatitis (AIH), who presented with sudden onset headaches from 3 weeks prior, and a single episode of seizure attack the morning before admission. In the initial examination she had highly elevated blood pressure (BP) (190/116). Her neurological examination revealed decline in force of limbs in addition to mild paresthesia. After primary stabilization, she underwent brain magnetic resonance imaging. Due to the clinical and radiological findings, the patient was diagnosed with PRES. In the following work-up of BP elevation, abdominopelvic sonography and subsequent computed tomography scan, multiple lesions were observed in spleen and both kidneys consistent with infarction. In further evaluation, Lupus-like anticoagulants were found to be elevated, which, in conjunction with the confirmed antiphospholipid syndrome (APS), suggested a possible role for APS-nephropathy as the missing link between PRES and APS. However, despite the role of an autoimmune disease in increasing the risk of developing other autoimmune conditions, APS and AIH have been rarely observed together. Our study indicates that developing APS in the context of AIH is a rare occurrence. However, APS could serve as a critical intermediary, potentially facilitating the onset of PRES despite lower BP.

## Introduction

Antiphospholipid syndrome (APS) is an autoimmune disease with systemic manifestations, including vascular thrombosis and pregnancy complications. The diagnosis is made by observing thrombotic complications or pregnancy morbidity in the presence of at least 1 positive laboratory criterion at a 12-week interval measurement based on the Sapporo criteria. Complications during pregnancy consist of repeated (3 or more) miscarriages in the first trimester, unexplained fetal death, and early delivery prior to the 34th week of pregnancy due to severe preeclampsia or placental insufficiency. The laboratory criteria consist of antiphospholipid antibodies (aPLs), including lupus anticoagulant (LA), anticardiolipin (aCL) antibody, and anti–β2glycoprotein-I (aβ2GPI) antibodies. However, many patients initially present with noncriteria manifestations [1].

Globally, APS is recognized as the most frequently acquired form of thrombophilia, with an estimated prevalence of 40-50 patients in 100,000 people. Studies on APS have indicated a survival rate of approximately 91% over a decade. From a clinical perspective, any organ can be impacted, including large, medium, or small vessels [2].

APS is frequently observed in patients with autoimmune diseases, such as lupus erythematosus. However, the simultaneous presentation of APS with autoimmune hepatitis is rarely described.

Autoimmune hepatitis (AIH) is a progressive immune-mediated disease of the liver, manifested by mostly nonspecific symptoms such as nausea, vomiting, fatigue, abdominal pain, or jaundice. As established by International Autoimmune Hepatitis Group (IAIHG), AIH diagnosis is based on increased autoantibodies, hypergammaglobulinemia, and distinctive histologic findings. However definite diagnosis is made after clinical assessment in addition to ruling out other causes including viral hepatitis [3]. The advancement of liver damage, potentially resulting in fibrosis and even cirrhosis, is due to the combined effects of an autoimmune response and other inflammatory factors. AIH is strongly associated with genetic susceptibility, triggered by environmental factors such as infections [4]. The exact etiology of AIH is not fully understood.

While affecting mostly women, AIH can occur in individuals of all ages and ethnicities. AIH has a prevalence ranging from 160 to 170 cases per 1,000,000 people in Europe, roughly equivalent to PBS. Without treatment, the prognosis for AIH can be severe, with 5 and 10-year survival rates dropping to as low as 50% and 10% respectively. AIH is often associated with a variety of other autoimmune diseases. These can manifest either in the patient themselves or in their first-degree relatives [5]. Basis of AIH treatment relies on steroids for inducing remission, followed by azathioprine as maintenance therapy [3].

Posterior reversible encephalopathy syndrome (PRES) is a neurological disease characterized by specific radiological findings of vasogenic edema, associated with a variety of neurological findings such as sudden onset headaches, seizures, visual disturbances, and encephalopathy. Neuroimaging criteria are essential for the diagnosis of PRES. Edematous focal regions in bilateral hemispheres of the brain are the most common radiological finding [6].

The most accepted hypothesis suggests a disruption in the blood-brain barrier as a result of a hypertensive crisis. The second theory suggests endothelial dysfunction, supported by the observation of PRES in patients with sepsis, eclampsia or preeclampsia, autoimmune diseases, or receiving immunosuppressive drugs despite normal BP [7].

In this investigation, we are going to present a 36-year-old female with a history of AIH who was referred with acute symptoms indicating PRES. In further assessment of neurologic symptoms and evaluating secondary causes for elevated BP, it was discovered that she had thrombotic lesions suspected to be APS, which was confirmed by antiphospholipid antibodies (Abs) in serology tests.

## Case presentation

A 36-year-old female with a history of autoimmune hepatitis (AIH) with a chief complaint of new-onset severe headaches in the frontal area, which had been present for 3 weeks before admission, was referred to Ayatollah Kashani Hospital, Isfahan, Iran. Accompanying the headaches, she experienced nausea and vomiting. Additionally, she reported paresthesia in both upper and lower limbs. The patient also reported an episode of losing consciousness and jaw-locking, which were reminiscent of a seizure attack in the morning prior to administration. Considering her known history of autoimmune hepatitis, the medical team suspected a potential onset of hepatic encephalitis or other major causes. Due to the severity and criticality of her symptoms, she was transferred to the Intensive Care Unit for continuous monitoring.

Her past medical history included an AIH confirmed by liver biopsy, which was performed 4 years ago. Notably, she had been taking azathioprine, prednisone, and metoprolol as part of her drug regimen.

In the general examination, the patient was well-oriented to time and place. Their temperature fell within the normal range. However, the blood pressure (BP) was highly elevated during the initial assessment (190/116 mmHg). Additionally, the patient demonstrated stage 2 hypertension (HTN) according to ACC/AHA guidelines [8]. The difference in BP between the 2 hands was negligible. Peripheral pulses were regular and symmetric. Due to the detection of elevated BP on most occasions, valsartan/hydrochlorothiazide was added to her drug regimen after initial stabilization.

At the time of the initial assessment, there was no tenderness in any area of the face, neck, or head, such as temporal tenderness or jaw claudication. Additionally, there was no pain or redor in the neck.

During the neurological examination, cranial nerves were intact. The force of the left upper and left lower extremities was mildly declined (4/5) as assessed by the muscle power scale of the Medical Research Council. The patient reported mild paresthesia in the upper/lower limbs during the initial assessment. Deep tendon reflexes (DTRs) were normal.

Complete blood count remained normal except for mild absolute neutrophilia. In the laboratory analyses of blood, the erythrocyte sedimentation rate (ESR) and C-reactive protein (CRP) levels were elevated. Additionally, aspartate aminotransferase (AST) and alanine transaminase (ALT) demonstrated only mild elevation compatible with the remission phase of AIH. Kidney function tests, serum electrolytes, and thyroid-stimulating hormones were normal (Table 1).

**Table 1:** Laboratory finding of our case.

Test	Result	Normal ranges	Test	Result	Normal ranges
BS (mg/dl)	114	70-140	Bili T (mg/dl)	0.8	0.1-1.2
BUN (mg/dl)	20	6-25	Bili D (mg/dl)	0.2	0.1-0.2
Cr (mg/dl)	0.8	0.6-1.3	AST (IU/L)	49	<31.0
Na (mEq/l)	137	136-145	ALT (IU/L)	59	<31
K (mEq/l)	4.4	3.8-5	ALP (IU/L)	159	70-260
RBC (Mil/mm^3^)	4.13	4.1-5.1	ANA	0.34	Neg < 0.8
Hb (g/dl)	12.5	12.3-15.3	ANCA –P	7.0	Neg <= 12
Hct (%)	37.4	35.9-44.6	ANCA–C	< 3.0	Neg <= 12
MCHC (g/dl)	33.4		Cardiolipin Ab	6.0	Neg < 12
Lymph (%)	20.2%	20-40	Anti–glut	Neg	
MIX (%)	3.2%	3-12	Anti–GAD	Neg	
PLT (/mm3)	287,000	150000-400000	Anti LGL 1	Neg	
ESR (mm)	24	0-20	CASPR 2	Neg	
TSH (mlu/L)	0.63	0.3-5.0	Anti DPPX	Neg	
Wright agglutination test	Neg		Anti-dsDNA (IF)	<1:10 (neg)	Neg < 1:10 Pos > 1:10
Anti-CCP Antibody	13.6	Neg<12 Pos>18 12<borderline<18	C.ANCA (IF)	< 1:20 (Neg)	Neg < 1:20 Borderline:1:20 Pos > 1:20
Coombs Write	Neg		P.ANCA (IF)	< 1:20 (Neg)	Neg < 1:20 Borderline:1:20 Pos > 1:20
Anti Cardiolipin; ACA IgG	1.39	Neg<12 Pos>18 12<borderline<18	ANA (IF)	< 1:20 (Neg)	Neg < 1:80 Pos > 1:80
Anti Cardiolipin; ACA IgM	4.36	Neg<12 Pos>18 12<borderline<18	Anti PR3 (C.ANCA)	5.06	Neg < 10 Pos > 10

ACA, anti-cardiolipin antibodies; ALT, alanine aminotransferase; ANA, antinuclear antibodies; Anti-GAD, anti–glutamic acid decarboxylase; AST, aspartate aminotransferase; BUN, blood urea nitrogen; CPK, creatine phosphokinase; Cr, Creatinine; CRP, C-reactive protein; DPPX, anti-dipeptidyl-peptidase-like protein 6; ESR, erythrocyte sedimentation rate; Hb, hemoglobin; Hct, hematocrit test; MCHC, mean corpuscular hemoglobin concentration; PLT, platelets; RBC, red blood cell; TSH, thyroid-stimulating hormone.

**Fig. 1:**
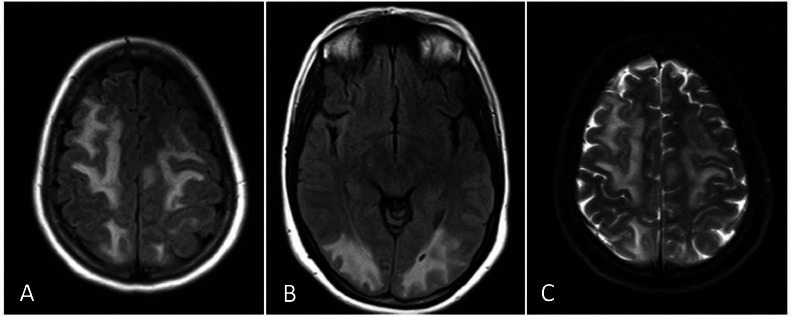
(A and B) Axial FLAIR (fluid-attenuated inversion recovery) and (C) Axial T2-weighted images show asymmetrical cortical, subcortical, and deep white matter with high signal intensity in fronto-parieto-occipital lobes.

**Fig. 2:**
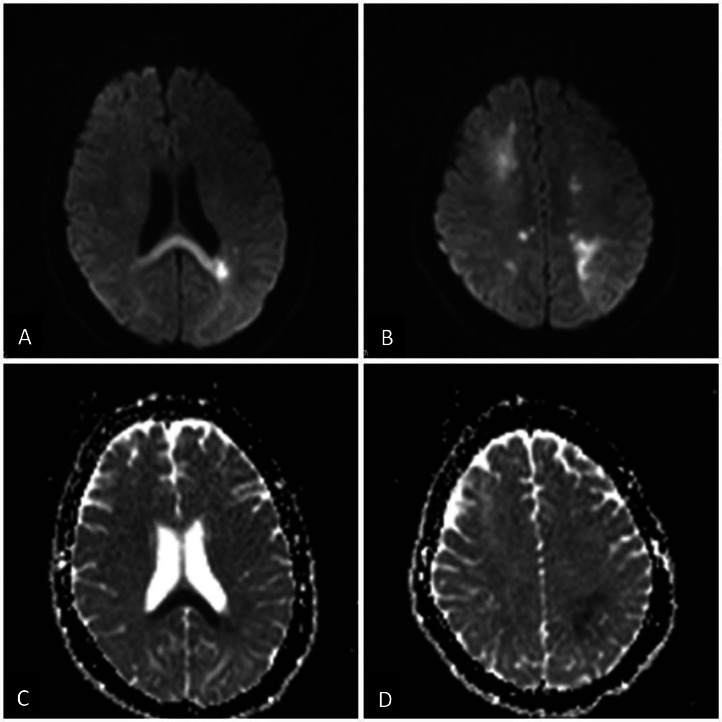
(A and B) DWI and (C and D) ADC maps images demonstrate true restriction in the corpus callosum and both centrum semiovale in favor of CLOCCs and multifocal acute infarction.

**Fig. 3:**
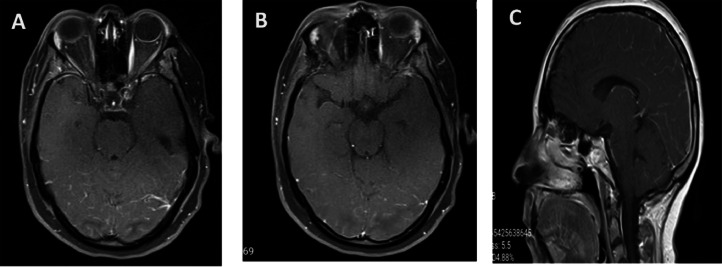
Brain MRI axial (A and B) and sagittal (C) Postcontrast images show leptomeningeal enhancement in parieto-occipital lobes, compatible with PRES.

Brain magnetic resonance imaging (MRI) was performed, and FLAIR (axial) and T2 (axial) images demonstrated asymmetrical cortical, subcortical, and deep white matter high signal intensity in the fronto-parieto-occipital lobes. Also, DWI and ADC images revealed actual restriction in the corpus callosum and both centrum semi-ovale, suggesting cytotoxic lesions of the corpus callosum (CLOCCs) and multifocal acute infarction. The occurrence of seizures and headaches, in conjunction with white matter lesions in fronto-parieto-occipital lobes in brain MRI, strongly suggests PRES as the main diagnosis (Fig. 1, Fig. 2). Also, postcontrast images showed leptomeningeal enhancement in parieto-occipital lobes (Fig. 3). Due to the severity of the clinical presentation, the patient was transferred to the intensive care unit (ICU).

Electrocardiograms showed normal sinus rhythm. Additionally, no abnormalities occurred during the heart rhythm monitoring with holter-monitoring.

Electroencephalography revealed abnormal paroxysmal slowing.

**Fig. 4:**
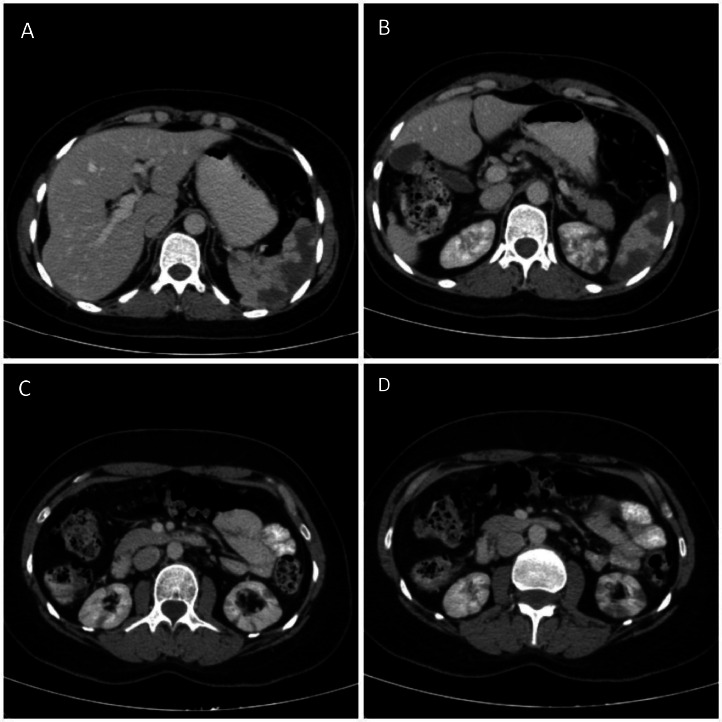
(A-D) Axial images of abdominopelvic CT scan with contrast show nonenhancing hypodense wedge-shaped areas in the spleen and both kidneys that favor multiple infarcts. Also, mild atrophy of the right liver lobe is depicted, secondary to autoimmune hepatitis.

During hospitalization, multiple episodes of HTN crisis were repeated. Accordingly, renal Doppler ultrasound was performed to rule out secondary etiologies for HTN. Renal arteries were intact on both sides. During abdominopelvic sonography for evaluating the liver, no abnormality was found, But hypoechoic areas were detected in both kidneys and spleen without vascularity as an incidental finding. Therefore, abdominopelvic contrast-enhanced computed tomography was performed, which revealed multiple wedge-shaped hypodense areas in both kidneys and spleen, consistent with infarction (Fig. 4).

Echocardiography did not reveal vegetation or other signs of endocarditis or an origin for brain emboli.

Coagulation tests were performed on the patient to evaluate the reason behind thrombophilia (Table 2). Partial thromboplastin time was in the normal range. Serology tests assessed Lupus-like anticoagulants (antiphospholipid antibodies) using the diluted Russell viper venom time (dRVVT) method, which was elevated, suggesting APS. Other serology tests were negative, as attached. Considering the hypercoagulate state, enoxaparine was added to the therapy plan. Notably, in the follow-up assessments 12 weeks later, the patient was positive for those tests.

**Table 2:** Coagulation tests of our case.

Coagulation department	Result	Normal ranges
aPTT	34	25-42
dRVVT screen test (s)	45.3 (H)	30.9-40.8 s
dRVVT confirm test (s)	43.1 (H)	27.8-38.2 s
dRVVT mixed test (1/2 + 1/2) (s)	30.5	
dRVVT screen ratio	1.36 (H)	<1.2
dRVVT confirm ratio	1.2 (H)	<1.2
dRVVT normalized test	1.29 (H)	<1.2

aPPT, activated partial thromboplastin time, dRVVT, diluted Russell viper venom time.

In summary, there were no signs of endocarditis or cardiac rhythm abnormalities responsible for renovascular/splenic lesions or neurological manifestations. Considering the clinical picture, APS appears to be the most probable diagnosis. Due to serology test results and multiple infarcted lesions, the patient met both clinical and laboratory criteria, so she was diagnosed with APS.

## Discussion

PRES is a neurological syndrome characterized by acute symptoms such as seizures, headaches, and focal neurological deficits. The pathophysiology relies on vasogenic edema due to blood-brain barrier dysfunction. Two theories are suggested to explain this phenomenon. The most widely accepted theory suggests that HTN is the causative agent. Accordingly, rapid elevations in BP exceed the endothelial autoregulatory potential in conditions such as hypertensive crisis [7,9].

With a prevalence of approximately 90%, HTN is the most common underlying condition in PRES, followed by systemic lupus erythematosus (SLE). Severe HTN and malignant HTN are observed in 31% and 12% of the APS patients, respectively. Despite the much lower prevalence of PRES in APS patients than in patients with SLE, the mechanism of developing PRES seems to share similarities due to the exact characteristics of both diseases [10].

Initially not defined in the Sapporo criteria introduced in 2006, APS-nephropathy was included in the classification criteria by the European League Against Rheumatism/American College of Rheumatology (EULAR/ACR) in 2023 [11].

APS-related BP elevation is considered primarily renovascular, as intravascular renal abnormalities, such as thrombotic microangiopathy, are the primary pathophysiological findings in biopsies. This elevation in BP, as a consequence of renal involvement, could manifest as new onset HTN or worsening of previously controlled HTN and other kidney-related symptoms. Consequently, renal involvement could be overlooked in developing PRES [10].

However, due to the silent progression of the disease, renal lesions often remain undiagnosed until more severe complications occur. Therefore, in most cases, renal lesions are discovered only as incidental findings revealing old silent infarctions on CT scans conducted for other purposes. Additionally, it could accompany ischemic end-organ injury to other systems, such as the central nervous system, cardiovascular and respiratory systems, and the gastrointestinal tract [[12], [13], [14]].

A second theory suggests brain endothelial dysfunction as an adverse effect of circulating toxins and cytokines. This is supported by the fact that 15%-20% of patients demonstrating PRES are normotensive or even hypotensive. Additionally, among hypertensive patients, up to 50% have a mean BP below the autoregulation capacity of the brain. Therefore, there should be another mechanism behind vasogenic edema in these patients. This theory is supported by the occurrence of PRES in patients with sepsis, pre-eclampsia, autoimmune diseases, or individuals receiving immunosuppressive drugs despite no significant rise in BP [7,9]. Thus, autoimmunity and the use of cytotoxic drugs could potentially facilitate the occurrence of more severe PRES. However, due to the remission phase of AIH, hepatic etiology is less proposed as a reason behind developing PRES, suggesting APS as the central pivot behind PRES.

Proper BP management, maintenance of kidney function, and discontinuation of causative substances are essential to successfully treating PRES related to HTN. However, due to underlying immune diseases in our case, withdrawing immunosuppressive drugs like corticosteroids seems debatable despite the possible long-term effects on BP. Thus, management in such cases relies mainly on tight control of BP [15]. Due to the thrombophilic characteristics of the disease, anticoagulant therapy is essential. In analyzing BP among APS patients, Sangle et al. [10] observed that receiving anticoagulant therapy leads to better control of BP and even reversal of renal and arterial lesions.

Radiological assessment can help to diagnose both PRES and APS. PRES is identified by bilateral vasogenic edema in the parieto-occipital region, often with a more limited distribution [16]. Conversely, APS is determined by a more widespread distribution of vasculitis and inflammatory changes, usually involving multiple organs such as kidneys, nervous system, and also peripheral vascular system [17].

The most frequently described abnormalities in PRES include symmetric cortical and subcortical hyperintensities on T2-weighted and FLAIR MRI in the parieto-occipital lobes of both hemispheres. These areas are often hypointense on corresponding T1-weighted MRI images and hypoattenuating on CT scans. Similar areas of altered signal intensity may also be seen in other locations, such as the frontal lobe, cerebellum, brainstem, and basal ganglia [18]. Additionally, contrast enhancement has been reported in PRES, often presenting as a leptomeningeal or gyral cortical enhancement [19]. According to McKinney et al. [20], enhancement was observed in 37.7% of the patients studied.

When we encounter a young patient with multiple infarcts in different areas, we suspect vasculitis, especially APS. On the other hand, when the patient also mentions symptoms of headache and high BP (like our patient), one of our differential diagnoses is PRES. In our patient, the suspicion of PRES and APS became more potent when we saw the brain images.

Similar to our study, Larmour et al. [21] reported that a 19-year-old female with a previous history of pancreatitis presented with abdominal pain, anuria, and vomiting. During the initial assessment, acute kidney injury was diagnosed for the patient secondary to pancreatitis. After initial stabilization, new-onset headaches and seizures were observed in the patient. In the brain, the MRI findings were compatible with PRES. However, the patient's BP did not exceed 150/90 during this time. After the initial discharge, she was readmitted due to an episode of seizure. In the renal biopsy, infarctive changes were observed to represent vascular thrombosis. The detection of phospholipid-dependent inhibitor antibody as a noncriterion laboratory finding and other negative serologic tests suggested APS as the most probable diagnosis responsible for PRES. In summary, they reported a rare case of PRES associated with secondary APS.

Interestingly, our case is among the rare cases that APS presents in the context of AIH. Although autoimmune disease history is common in AIH and APS patients, their association is rarely reported. Noteworthy aPLs have been frequently observed in patients with infectious hepatitis. However, due to the absence of thrombotic manifestations, they did not fulfill the criteria for APS, bringing up the theory that these antibodies are secondary to the body's natural response to the destruction of hepatic cells [22]. Therefore, the occurrence of APS in the context of AIH remains uncommon [23,24]. It is noteworthy to mention that, although rare, APS can sometimes present prior to the onset of AIH [25,26].

In 2001, Dourakis et al. [24] reported 2 cases of APS following AIH. They reported a 31-year-old female with a history of AIH from 6 years before referral and a pregnancy loss prior to administration. The patient has received prednisolone and azathioprine. However, azathioprine was discontinued due to pregnancy. In the initial examination, the patient demonstrated icteric sclera and hepatomegaly. In the laboratory assessment, AST was 730 IU/l, and ALT was 556 IU/l, in addition to hyperbilirubinemia. Additionally, ESR was elevated (56 mm/h). In Coagulation studies, prolonged prothrombin and activated partial thromboplastin times were elevated at 14.8 s and 62.4 s. In serum protein electrophoresis, albumin was decreased, and an ã-globulin was increased (28%). The lupus anticoagulant and IgG anticardiolipin antibodies were positive, consistent with APS. Eventually, the physical examination and laboratory findings confirmed APS, which is secondary to AIH.

In summary, we presented a female with a history of AIH who developed PRES, initially presumed to be only secondary to HTN. In further investigations, it was revealed that she had APS and subsequent nephropathy, facilitating the occurrence of PRES.

## Conclusion

In this unprecedented case, we reported the coexistence of AIH, APS, and PRES in a single patient. Our study indicates that developing APS in the context of AIH is a rare occurrence. However, APS could serve as a critical intermediary, potentially facilitating the onset of PRES despite lower BP. The insidious progression of the disease often results in asymptomatic phases, with clinical manifestations becoming apparent only upon the development of more advanced complications.

Furthermore, we concluded that whether remaining undiagnosed or receiving immunosuppressive drugs, underlying autoimmune diseases could potentially render patients susceptible to vasogenic edema, which is a fundamental pathophysiological mechanism responsible for developing PRES.

## Patient consent

Complete written informed consent was obtained from the patient for the publication of this study and accompanying images.
